# Comparative analysis of the fecal microbiota in Père David's deer and five other captive deer species

**DOI:** 10.3389/fmicb.2025.1547348

**Published:** 2025-03-26

**Authors:** Caiquan Zhao, YuChen Yang, Peng Zhao, LiGe Bai

**Affiliations:** College of Ecology and Environment, Baotou Teacher's College, Baotou, China

**Keywords:** Père David's deer, gut microbiota, 16S rRNA gene, metabolic pathways, conservation

## Abstract

**Introduction:**

Gut microbes are essential for host nutrition, immunity, and development. Various factors influence the composition and function of the gut microbial community. However, there is limited knowledge regarding the comparison of gut microbiota across different deer species, particularly those in the World Deer Park of Baotou (Inner Mongolia, China).

**Methods:**

This study utilized 16S rRNA gene amplicon sequencing to analyze the fecal microbiota and potential microbial function in Père David's Deer (*Elaphurus davidianus*), Sika deer (*Cervus nippon*), American Wapiti (*Cervus canadensis*), Red Deer (*Cervuselaphus*), Fallow Deer (*Dama dama*), and Reindeer (*Rangifer tarandus*).

**Results and discussion:**

The findings indicated no significant differences in alpha diversity, yet there was a noteworthy distinction in beta diversity among the six deer groups. At the phylum level, the predominant bacteria in the deer populations were Firmicutes, Bacteroidetes, and Proteobacteria. At the genus level, 54 core bacterial microbiota were identified. The top four genera in AW, FD, PD, and SD were Ruminococcaceae UCG-005, Rikenellaceae RC9 gut group, RuminococcaceaeUCG-010 and Christensenellaceae R-7 group. The results of the neutral model revealed that neutral processes predominantly governed the gut microbiota community assembly in different deer species, particularly in Père David's deer. PICRUSt2 predictions showed significant enrichment of fecal bacterial functions related to fatty acid, lipid, metabolic regulator, and amino acid biosynthesis. This comparative analysis sheds light on the microbial community structure, community assembly, and potential functions, offering improved insights into the management and conservation of deer species, especially Père David's deer. Future research might focus on exploring metagenomic functions and dynamics in wild settings or across different seasons using metagenomics or metatranscriptomics.

## Introduction

The Père David's deer (*Elaphurus davidianus*, Milu in Chinese) is endemic to China and is listed on the IUCN Red List as Extinct in the Wild (Jiang and Harris, 2016). It was once widely distributed in the middle and lower reaches of the Yangtze River, China. Père David's deer was first introduced to European in 1866 by Armand David (Père David). This species became extinct in the wild in China in the early twentieth century (Keqing, 1985). Fortunately, surviving deer prospered in Britain and formed the basis for reintroduction to China in 1985 (Jiang et al., 2000). Initially, 77 descendants were reintroduced into China in captivity, but today, there are more than 6,000 Père David's deer spread over 70 regions throughout the country. However, the wild population of Père David's deer is still smaller than that of other deer populations. Père David's deer remains the national first-level protected species in China. As the Père David's Deer population increases, disease incidence has also risen, which has been proposed to be related to infections of the gastrointestinal tract (Bahrndorff et al., 2016; Yang et al., 2016a; Zhang et al., 2018). Therefore, further studies are required to focus on the diet composition, metabolism, and energy absorption of the Père David's deer for better conservation.

The gut microbiota of mammals has been increasingly recognized as a key factor affecting host health, nutrition, development, and productivity (Muegge et al., 2011; Holmes et al., 2012; Yeoman and White, 2014). Gut microbiota has a symbiotic relationship with their hosts and is an integral part of animals (Adak and Khan, 2019). Host behavior has an impact on symbiotic gut microbiota, including microbial community formation, regulation of microbial composition at different life history stages, and active control of microbial populations, and facilitates a variety of physiological activities for the host, such as nutrient absorption, metabolism, and immunity (Ezenwa et al., 2012; Andoh, 2016). Unlike monogastric animals, ruminant guts have a complex ecosystem, and bacteria are closely related to the health of ruminants (Malmuthuge and Guan, 2017). Numerous studies have confirmed that bacteria such as Pasteurella, pathogenic *Escherichia coli, Clostridium septicum*, and *Clostridium perfringens* are a serious threat to the survival of the deer, and they normally infect Père David's deer in combination (Yapin, 1986; Qiu et al., 2014; Fitzgerald et al., 2023; Prentice et al., 2024). Therefore, studying the composition and structural characteristics of the gut microbiota in different deer species allows us to assess and ensure their health status and provide scientific guidance for the captive conservation of Père David's deer.

The significance of identifying the gut microbial community is that it provides the scientific basis for protecting endangered animals. Previous studies have shown that the environment, genetics, disease, and diet can affect the balance and health of the gut microecology (Moeller et al., 2013; Waite et al., 2014; Kers et al., 2018). The diet of Père David's deer varies in different breeding areas and environments (Zhang et al., 2018). The gut microbiome of Père David's deer in Dafeng potentially coevolved with the host diet, reflecting local adaptation of the translocated population in their new living environment (Wang et al., 2019). Furthermore, a recent study indicated stark differences in the gut microbial community composition between wild and captive Père David's deer in Dafeng Nature Reserve (Sun et al., 2019a). Today, captives are an effective conservation strategy that decreases the risk of death by increasing the Père David's deer population. Therefore, it is urgently necessary to understand the composition and function of the gut microbiota in Père David's deer to provide an avenue for exploring the potential exchange of microbiota between different deer species and breeding areas.

Here, we performed a comprehensive analysis of the fecal bacterial microbiota of six captive deer species (raised in the same location with same diet), including Pere David's Deer (*Elaphurus davidianus*), Sika deer (*Cervus nippon*), American Wapiti (*Cervus canadensis*), Red Deer (*Cervus elaphus*), Fallow Deer (*Dama dama*), and Reindeer (*Rangifer tarandus*), covering two national first-level protected species in China and four common species at the World Deer Park of Baotou (Inner Mongolia, China). Fecal microbial composition was identified by 16S rRNA gene amplicon sequencing and microbiota function was predicted using PICRSUt2. The purpose of this study was to determine the fecal microbial community composition and compare the potential microbial function among deer species. This study enhances our understanding of the gut microbiota in different deer species and provides scientific data for Pere David's deer conservation.

## Materials and methods

### Study objects and sample collection

All 30 fresh fecal samples were collected from six captive deer species, including the national first-level protected species in China, such as Pere David's Deer (*Elaphurus davidianus*) and Sika deer (*Cervus nippon*); common species of American Wapiti (*Cervus canadensis*), Red Deer (*Cervus elaphus*), Fallow Deer (*Dama dama*), and Reindeer (*Rangifer tarandus*) at the World Deer Park of Baotou (Inner Mongolia, China) in winter (Supplementary Table 1). To prevent soil contamination, only the middle layer of feces was collected from these individuals. All sampled deer were adults, had not recently been treated with antibiotics, and were confirmed to be in good health. Samples were immediately transferred to 5-mL sterile tubes and stored in liquid nitrogen. After collection, the samples were sent to the laboratory and stored at −80°C until further processing.

### DNA extraction and sequencing

Genomic DNA was extracted from the fecal samples using the CTAB method (Honore-Bouakline et al., 2003). The extracted DNA was analyzed using a NANODROP LITE spectrophotometer (Thermo Scientific, USA) to evaluate DNA quantity and quality. We used the universal primers (341F [5′-CCTAYGGGRBGCASCAG-3′] and 806R [5′-GGACTACNNGGGTATCTAAT-3′]) to amplify the 16S rRNA gene V3–V4 region with a 6 bp barcode unique to each sample. PCR was performed with 15 μL of Phusion High-Fidelity PCR Master Mix (New England Biolabs), 0.2 μM forward and reverse primers, and 10 ng template DNA. The PCR conditions were as follows: initial denaturation at 98°C for 1 min, followed by 30 cycles of denaturation at 98°C for 10 s, annealing at 50°C for 30 s, and extension at 72°C for 30 s, and a final extension step at 72°C for 5 min. The PCR products were pooled and purified using a Qiagen Gel Extraction Kit (Qiagen, Germany). The Illumina TruSeq^®^ DNA PCR-Free Sample Preparation Kit (Illumina, United States) was used to produce sequencing libraries according to the manufacturer's recommended protocol. After detection of library quality, samples were sequenced using the Illumina NovaSeq platform in 250 bp paired-end running mode. The sequencing service was provided by Novogene Co. Ltd. (Tianjin, China).

### Sequencing data processing

All raw paired-end sequences were imported into the QIIME2 pipeline (version 2020.8.0) (Bolyen et al., 2019). Primers were removed using the Cutadapt plugin by “qiime cutadapt trim-paired” (-p-minimum length 200). The DADA2 plugin (“qiime dada2 denoise-paired”) was used to generate denoised feature sequences (amplicon sequence variants, ASVs) and feature tables (-p-trim-left-f 0 –p-trim-left-r 0 –p-trunc-len-f 235 –p-trunc-len-r 220) (Callahan et al., 2016). Feature sequences with a frequency ≤ 4 were discarded using “qiime feature-table filter-features–p-min-frequency 4.” Reference sequences were extracted from the SILVA database (release 132) using specific primers for the 16S V3–V4 region using “qiime feature-classifier extract-reads–p-min-length 200 –p-max-length 500” (Quast et al., 2013). The Naive Bayes classifier was trained for taxonomic annotation using the command line of “qiime feature-classifier fit-classifier-naive-bayes.” ASVs assigned to mitochondria and chloroplasts were excluded from the feature table (“qiime taxa filter-table–p-exclude mitochondria, chloroplast”) and feature sequences (“qiime taxa filter-seqs–p-exclude mitochondria, chloroplast”). We used PICRUSt2 (Phylogenetic Investigation of Communities by Reconstruction of Unobserved States) software (https://github.com/picrust/picrust2) to predict the functional abundance of the microbiota (Douglas et al., 2020).

### Statistical analysis

Alpha diversity indices of microbiota (Ace, Chao1, Simpson, goods_coverage, and Shannon index) were calculated using the command line of “qiime diversity alpha.” These indices were compared between groups using the non-parametric Wilcoxon rank-sum test. A principal coordinate analysis (PCoA) plot was generated based on the Bray-Curtis distance. Permutational analysis of variance (PERMANOVA) was applied to test group differences based on the Bray-Curtis distance matrix using the vegan package (Oksanen et al., 2007). Linear Discriminant Analysis Effect Size (LEfSe) was used to test differences in taxa abundance (LDA score ≥ 3.0, *p* < 0.05) and functional abundance (LDA score ≥ 3.0, *p* < 0.05) (Segata et al., 2011). The Sloan neutral community model has been used to assess the importance of stochastic processes in gut microbiota assembly (Sloan et al., 2006). Being derived from Hubbell's neutral theory (Hubbell, 2001), Sloan's model can recognize the competitive status of a species in a community and is suitable for assessing a large population size such as the microbiota community. Observed OTU distributions and mean relative abundances in each of the five populations were fit to this model using R code6, respectively (Burns et al., 2016). To run these scripts, packages, including remotes, Hmisc, devtools, mle, stats4, phyloseq, and DanielSprockett/reltools, were installed and loaded. Logistic regressions were performed using the presence/absence of taxa and partition types to identify taxa above or below the indicated partitions. The average abundance of each OTU across all caterpillar individuals in a population was fitted to the neutral model using the parameter of migration rate (m), and the fit of m for each population was assessed with a generalized R-squared. Taxa within 95% confidence intervals were considered well predicted by the neutral model. To test the difference in composition between the above and below partitions of the neutral model, distance-based redundancy analysis was conducted on the Jaccard indices (Heys et al., 2020; Li et al., 2022b).

## Results

### Diversity of fecal microbial communities of six deer species

**Figure 1:**
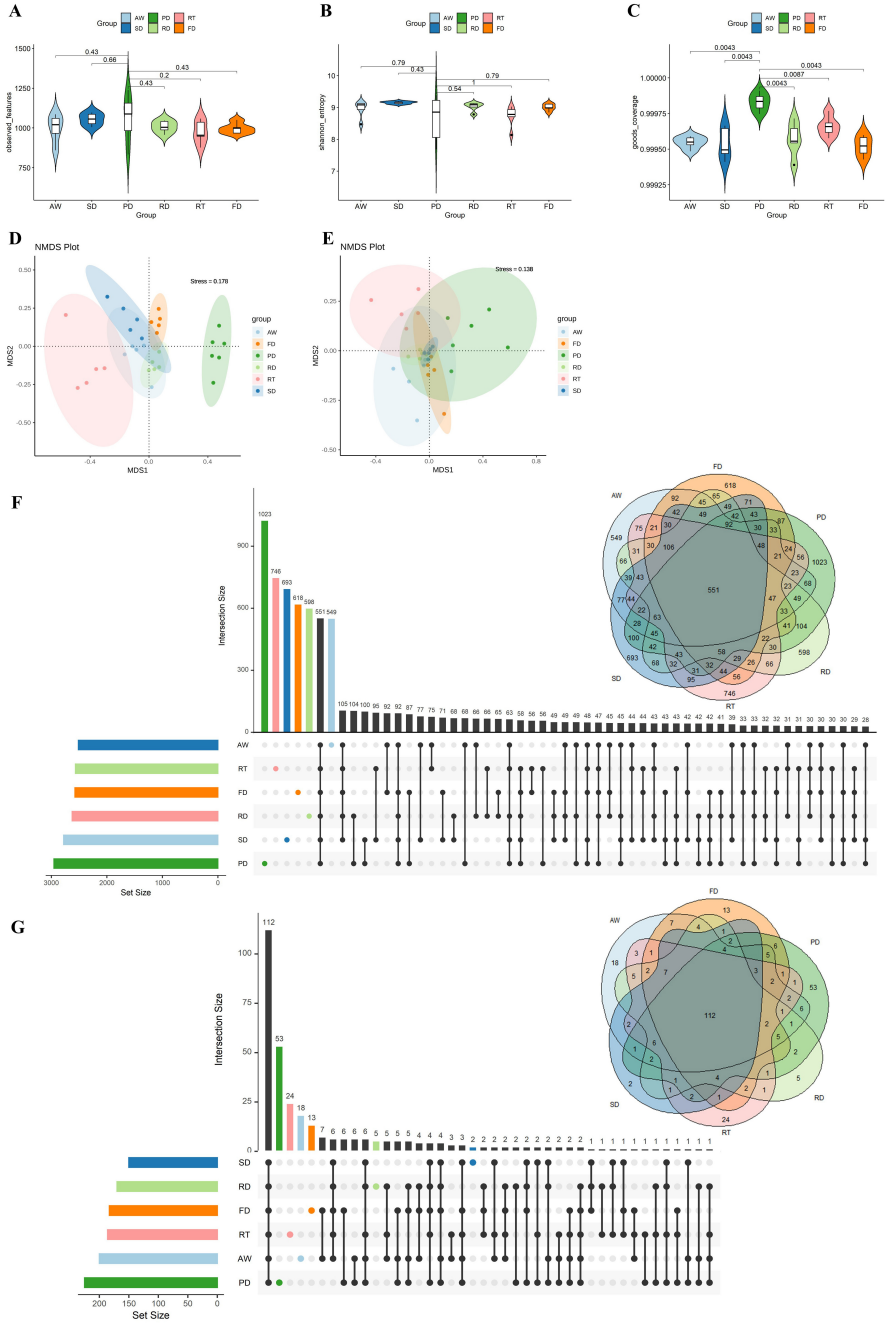
Alpha and beta diversities of fecal microbiota among the six deer species. Violin plots depicting differences in ASV richness among different deer species using the observed features index **(A)**, Shannon index **(B)**, and goods coverage index **(C)**. Non-metric multidimensional scaling analysis (NMDS) based on the Bray–Curtis dissimilarity matrix. This visualization demonstrates the dynamic changes in the gut microbiota composition of different deer species at the ASV **(D)** and genus **(E)** levels. Venn diagram of ASVs and genera overlapping across six deer species based on ASV **(F)** and genus **(G)** presence and absence.

After quality control, 1,939,525 valid sequences were obtained from all samples, averaging 62,565 ± 8,444 (mean ± SD) per sample (Supplementary Table 2). The rarefaction curve demonstrated that we achieved sufficient sequence depth to accurately characterize the fecal bacterial community in deer, as reflected by the number of observed features, as well as the Shannon and goods coverage indices (Supplementary Figure 1). To assess the diversity of fecal microbiota among different deer species, we conducted an alpha diversity analysis using various indices, including observed features, Shannon, goods coverage, ACE, Simpson, and Chao1 indices (Figures 1A–C and Supplementary Figure 2). The results indicated that alpha diversity did not significantly vary among the six deer species. Despite this lack of significant difference in alpha diversity, non-metric multidimensional scaling (NMDS) analysis based on ASV- and genus-level data revealed species-specific diversity of fecal microbial communities, using Bray-Curtis dissimilarity (Adonis, *p* = 0.001) (Figures 1D, E). Moreover, Venn diagram and UpSetR analysis showed that different deer species harbor unique enrichments of gut microbiota at the ASV and genus levels (Figures 1F, G). Notably, Père David's deer exhibited a remarkable 53 genera of gut microbiota unique to its species.

### Comparison of fecal microbial communities of six deer species

**Figure 2:**
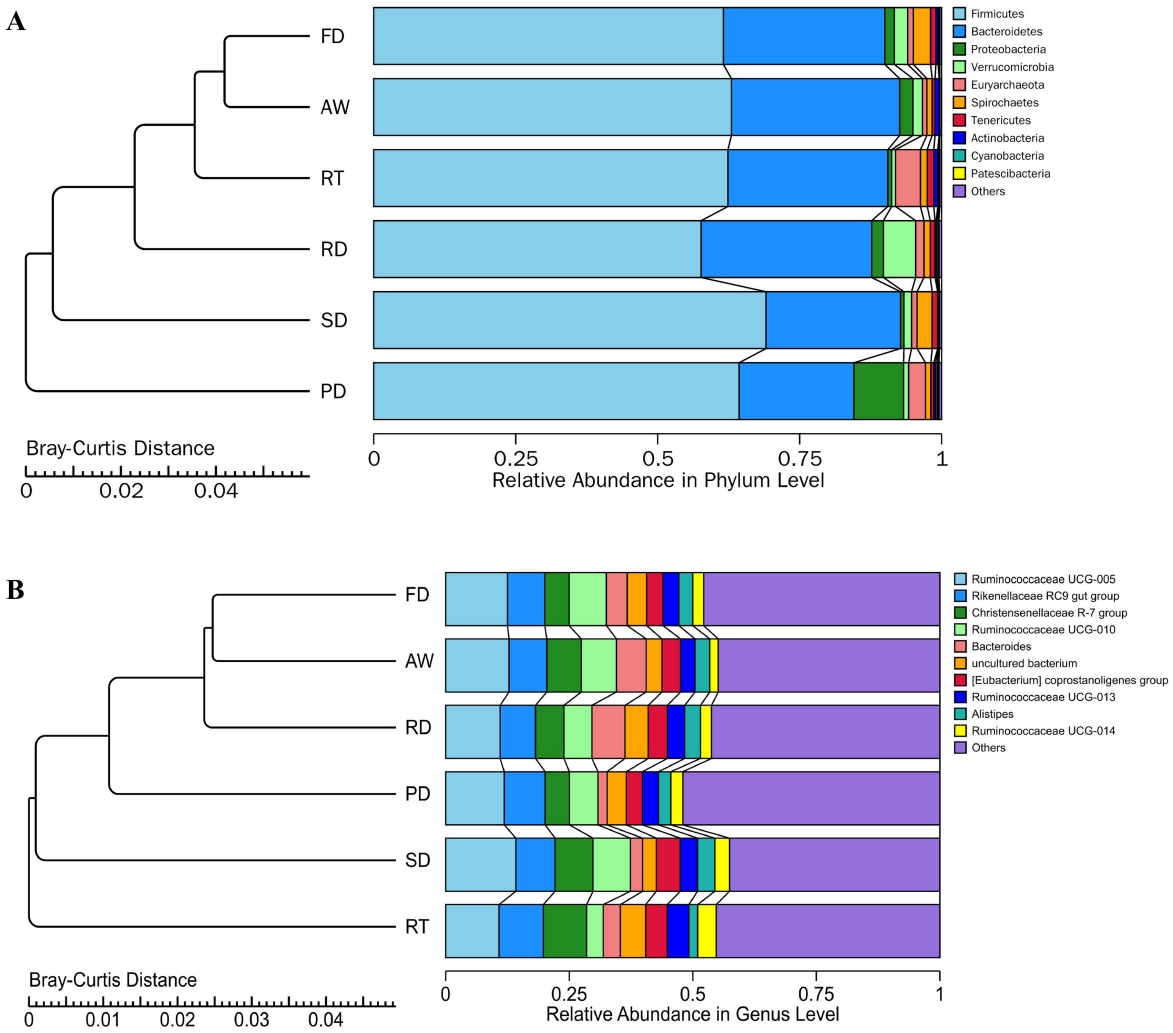
Bar chart of relative abundance. The bar chart **(right)** shows the relative abundance of the bacterial phyla **(A)** and genera **(B)** in each deer. The tree **(left) (A, B)** shows the hierarchical clustering of the samples based on Bray-Curtis dissimilarity.

All ASVs were classified into 24 phyla, 41 classes, 83 orders, 135 families, and 322 genera, including unclassified entries. We examined the bacterial composition of fecal samples at both the phylum and genus levels. Among the identified phyla in the fecal microbiota of six deer species, the predominant groups, constituting over 98% of the total bacteria, were Firmicutes (63%), Bacteroidetes (26.69%), Proteobacteria (2.68%), Verrucomicrobia (2.10%), Euryarchaeota (1.93%), and Spirochaetes (1.65%) (Figure 2A; Supplementary Table 3). The abundance of Proteobacteria was significantly higher in the PD group compared to other groups, while Bacteroidetes showed the opposite trend. At the genus level, we identified 54 core bacterial genera with an average relative abundance exceeding 0.2%. In the AW, FD, PD, and SD groups, the top four genera were Ruminococcaceae UCG-005 (12.83%, 12.52%, 11.87%, and 14.21%, respectively), Rikenellaceae RC9 gut group (7.63%, 7.55%, 8.27%, and 7.94%), Ruminococcaceae UCG-010 (7.11%, 7.55%, 5.79%, and 7.57%), and Christensenellaceae R-7 group (6.98%, 4.90%, 4.90%, and 7.67%) (Figure 2B). The RD group was dominated by Ruminococcaceae UCG-005 (11.02%), Rikenellaceae RC9 gut group (7.15%), Bacteroides (6.69%), and Christensenellaceae R-7 group (5.75%), while in the RT group, the leading genera were Ruminococcaceae UCG-005 (10.81%), Rikenellaceae RC9 gut group (8.96%), Christensenellaceae R-7 group (8.76%), and Ruminococcaceae UCG-013 (4.38%) (Figure 2B). Among the core genera, Succinivibrio, Paenibacillus, and Anaerovibrio were significantly more enriched in the PD group compared to other groups, whereas Bacteroides and Enterococcus showed a significant depletion in PD compared to their abundance in the AW, FD, and RD groups.

### NCM described the frequency distributions of gut microbial communities in difference deer species

**Figure 3:**
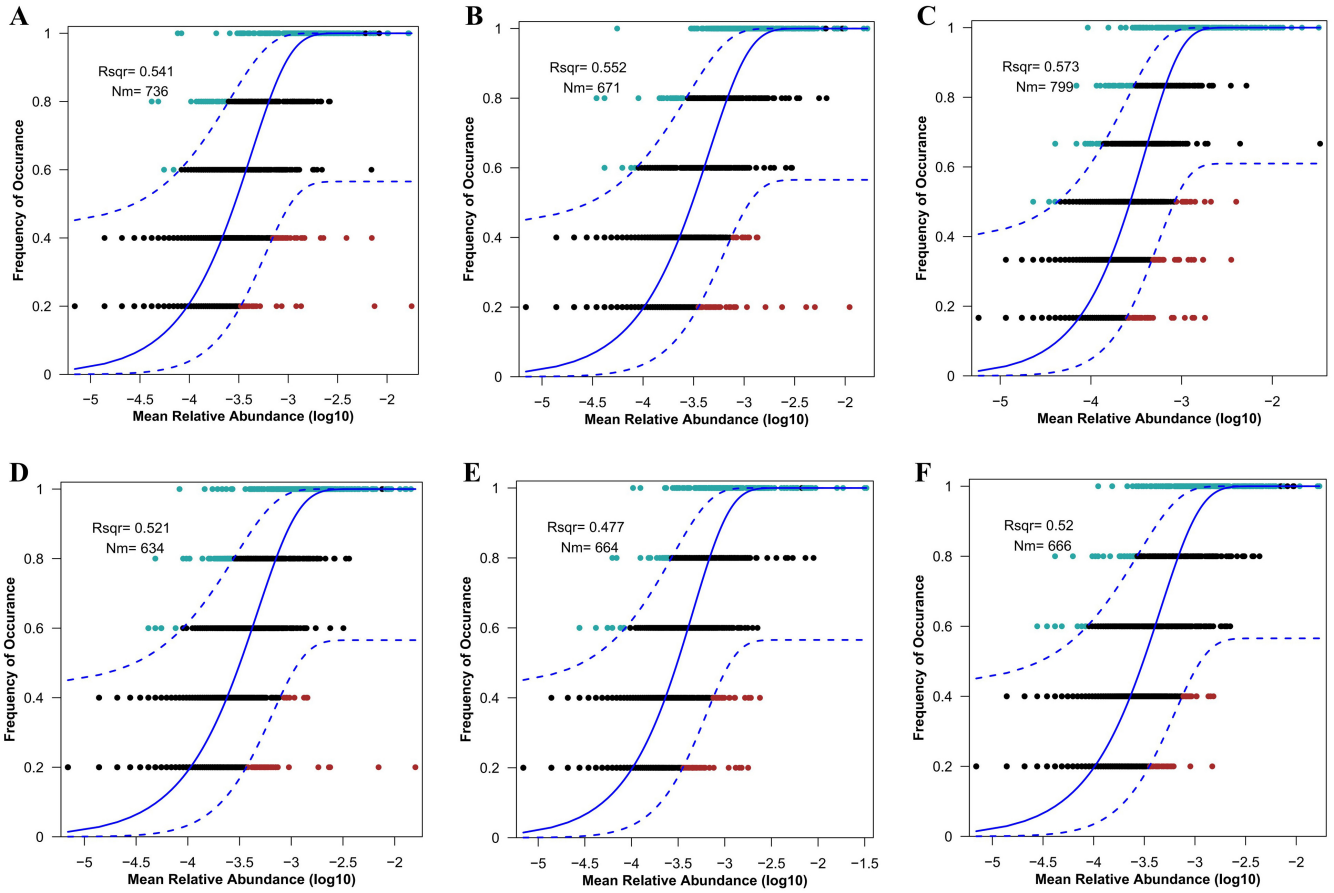
Fit of the neutral model to the gut microbial community. Goodness of fit of the neutral model for AW **(A)**, FD **(B)**, PD **(C)**, RD **(D)**, RT **(E)**, and SD **(F)**. This model provides estimates of ASVs occurrence given its abundance according to the best-fit neutral model, represented by a solid curve, and the dashed lines represent the 95% confidence intervals around the best-fitting neutral model. Each point in **(A–F)** represents an ASV in fecal bacteria. The R2 value indicates the goodness of fit of the neutral model. The value ranged from 0 (no fit) to 1 (perfect fit). Nm indicates the product of metacommunity size (N) and migration rate (m), quantifies estimates of dispersal between communities, and determines the correlation between occurrence frequency and relative regional abundance.

Variations in NMDS diversity among the gut microbial communities of deer may be attributed more to neutral processes than to deterministic ones. To evaluate the significance of neutral processes in the assembly of the deer gut microbiome, we applied a neutral model to the dataset. The frequency distributions of most ASVs within each deer population conformed to the predictions of the neutral model (Figures 3A–F). The PD group (57.3%) exhibited a greater relative contribution from stochastic processes compared to the FD (55.2%), AW (54.1%), RD (52.1%), SD (52%), and RT (47.7%) groups. A higher R^2^ value in the NCM not only indicates a better fit of the model to the gut microbial community data but also underscores the greater role that neutral processes play in shaping the community.

### Difference in fecal microbial communities of six deer species

**Figure 4:**
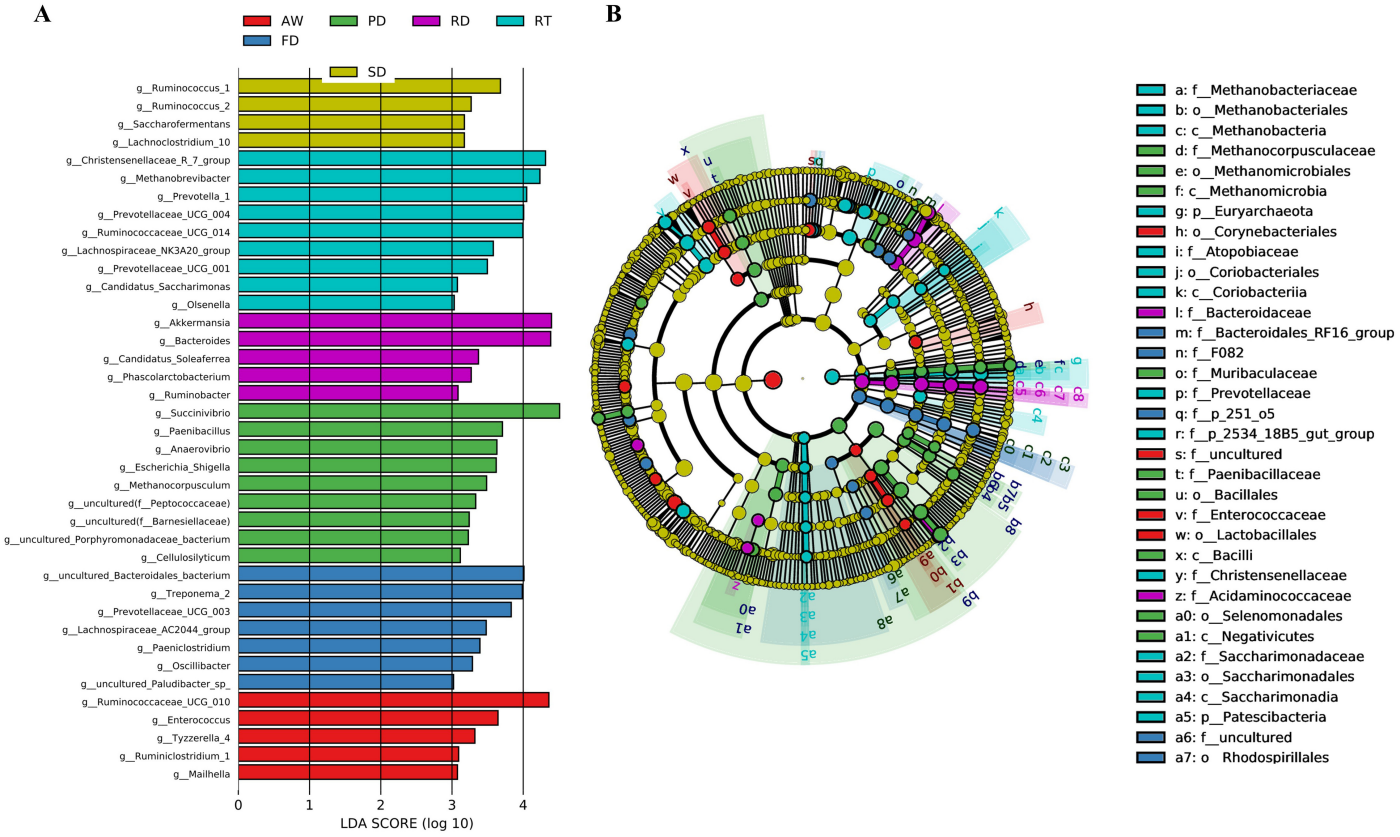
Linear discriminant analysis (LEfSe) effect size analysis. Differentially abundant bacteria were determined by LEfSe analysis using the Kruskal-Wallis test (*P* < 0.05) with an LDA score > 3 **(A)**. The cladogram showed microbial species with significant differences among the six groups. Different colors indicate different groups, with the species classification at the phylum, class, order, family, and genus levels shown from the inside to the outside **(B)**. The yellow nodes indicate that groups of gut microbes do not play a significant role.

To further determine the microbial taxa that best explained the differences between the six deer species, we conducted a LEfSe analysis (Figure 4A). The results indicated that in the SD group, *Ruminococcus, Lachnoclostridium*, and *Saccharofermentans* had significantly higher abundances compared to the other groups. In the RT group, *Olsenella, Prevotellaceae, Prevotella, Ruminococcaceae, Methanobrevibacter, Lachnospiraceae, Candidatus saccharimonas*, and *Christensenellaceae* were significantly more prevalent. *Phascolarctobacterium, Bacteroides, Candidatus Soleaferrea, Ruminobacter*, and *Akkermansia* were also more abundant in this group. The PD group showed significantly higher levels of *Escherichia_Shigella, Cellulosilyticum, Paenibacillus, Methanocorpusculum, Anaerovibrio*, and *Succinivibrio* than the other groups. Furthermore, the FD group contained a greater diversity of unique bacteria, including *Paeniclostridium, Treponema, Oscillibacter*, and *Lachnospiraceae*. In the AW group, Mailhella, Ruminococcaceae, Ruminiclostridium, Enterococcus, and Tyzzerella were notably more abundant (LDA > 3.0, *P* < 0.05). The cladogram highlighted significant differences in the gut microbial composition across the different deer species (Figure 4B).

### Potential function of the fecal microbiota of six deer species

**Figure 5:**
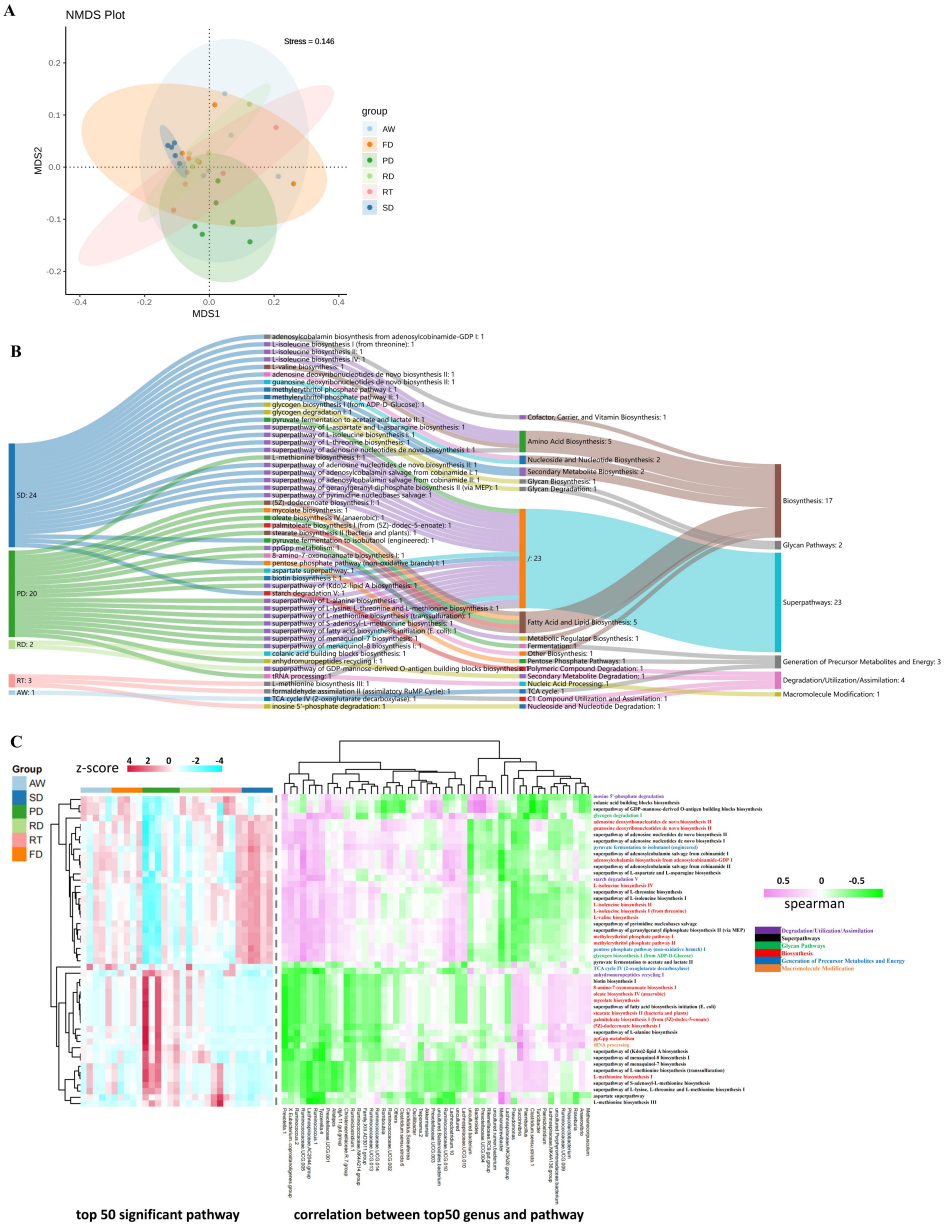
Prediction of microbial function. Non-metric multidimensional scaling (NMDS) ordinations based on Bray–Curtis distances and Bonferroni based on ANOSIM in the fecal microbiota of six deer species (stress value = 0.146) **(A)**. Sankey diagram delineating the top 50 significant pathways in the LEfSe analysis of different deer species **(B)**. Heatmap showing correlations between the top 50 genera of bacteria and KEGG function pathways in the six groups **(C)**. Clustering analysis was performed using Pearson's correlation and Euclidean distance based on the relative content of metabolic pathways and genera of bacteria. Data were processed using *z*-score transformation.

Based on the PICRUSt2 function prediction results, the functional abundance of Pathway, COG, EC and KO levels was analyzed by non-metric multidimensional scaling (NMDS) of fecal microbiota of six deer species (Supplementary Figure 3). The results indicated a distinct separation of EC-Pathway biomarkers for the PD and SD groups from the other four groups (Figure 5A). Additionally, among the top 50 differential biomarkers, the PD group (24 pathways) and SD group (20 pathways) exhibited a significantly higher number of enriched differential pathways compared to the other groups (Figure 5B). In the PD group, the predominant functional pathways included fatty acid and lipid biosynthesis, metabolic regulator biosynthesis, l-alanine biosynthesis, l-methionine biosynthesis, and other amino acid biosynthesis pathways. In contrast, the SD group showed enrichment in pathways related to amino acid biosynthesis, nucleoside and nucleotide biosynthesis, secondary metabolite biosynthesis, and several super pathways. Furthermore, correlation analysis was conducted between the top 50 KEGG functional pathways identified by LEfSe analysis and the relative abundance of the top 50 genera (Figure 5C). The results revealed that in the PD group, the enriched functional classes were primarily super pathways and those involved in the biosynthesis of fatty acids and lipids. The SD group was characterized by enrichment in super pathways, amino acid biosynthesis, precursor metabolite generation, and energy pathways. The other deer species also showed enrichment in several pathways shared with the PD and SD groups.

## Discussion

Interest in the fecal bacterial microbiota of animals has surged due to its crucial role in nutrition, health, development, and productivity. Both the host species and living environment have been suggested as major factors modulating the gut microbial composition of mammals. Père David's deer, a highly endangered species native to China, is often kept in captivity with the goal of reintroducing it into the wild. Therefore, we selected six deer species, including Père David's deer, to investigate differences in their fecal microbial communities.

In this study, we employed 16S rRNA Illumina MiSeq high-throughput sequencing technology to compare the core fecal microbiota of Pere David's Deer, Sika deer, American Wapiti, Red Deer, Fallow Deer, and Reindeer and analyzed the diversity of their microbial communities. Although these deer live in the same captive environment and consume a commercial corn-soybean basal diet, their gut microbial compositions are distinct. Our observations showed that while alpha diversity was similar, there were significant differences in NMDS diversity, indicating consistent species richness and evenness within the fecal microbial communities among the six deer groups, yet substantial diversity in their fecal bacterial profiles. Our data revealed that Firmicutes, Bacteroidetes, Proteobacteria, and Verrucomicrobia were the predominant phyla. Firmicutes were mainly represented by *Clostridia, Mollicutes, Thermolithobacteria, Bacilli, Negativicutes*, and *Erysipelotrichia* with *Clostridium, Roseburia, Lactobacillus, Ruminococcus*, and *Faecalibacterium* being the most studied genera (Jandhyala et al., 2015; Ndeh and Gilbert, 2018). These genera not only supply energy to hosts through the metabolism of sugars, fatty acids, and carbohydrates, but they also play a critical role in human homeostasis by degrading dietary fiber (Flint et al., 2008; Tap et al., 2009; Sun et al., 2023). The main function of Bacteroides is the metabolism of polysaccharides and bile acids, aiding in protein synthesis and enhancing host immune function (Hooper, 2004; Bäckhed et al., 2005). It has been reported that the richness of Bacteroides in the Bacteroidaceae family decreases in the gut microbiota of Père David's deer after consuming a soybean-rich diet (Zhang et al., 2018). Consistent with previous findings, our research showed that the Bacteroidetes in the gut microbiota of Père David's Deer have lower relative abundances compared to other deer, possibly due to their soybean-rich diet. Additionally, Studies have shown that high-starch and fat diets can lead to a considerable increase in the abundance of Proteobacteria in the intestinal tract of Sichuan Alpine musk deer, rabbits, mice, and obese children (Zhu et al., 2015; Méndez-Salazar et al., 2018; Jeong et al., 2019; Zhang et al., 2023). Previous studies have found that carnivorous raptors, considered pathogen vectors, have high relative abundances of Proteobacteria in their intestinal flora (Zhou et al., 2020; Zhao et al., 2022). Many microbes within Proteobacteria are known zoonotic pathogens, such as *Escherichia, Shigella, Salmonella*, and *Klebsiella*, which can cause intestinal diseases in animals or diarrhea in humans (Kotloff et al., 2013; Nyaoke et al., 2020; Yan et al., 2021). Pathogens associated with *Enterobacteriaceae*, a family within Proteobacteria, were found to predominate in the gut microbiota of Père David's deer. *Shigella*, a group of gram-negative bacteria that cause bacillary dysentery in humans and primates, was notably prevalent (Qasim et al., 2022). These findings collectively suggest that Père David's deer may suffer from intestinal inflammation. In the fecal microbiota of other deer species, phyla such as Tenericutes, Actinobacteria, and Cyanobacteria were identified as commensal bacteria, primarily influenced by diet. Referring to the above results, it is clear that different dietary preferences have a significant impact on the diverse gut microbial composition of different deer species. Although captives are an effective conservation strategy for Père David's deer population recovery, some potential health risks cannot be ignored, such as reduced cellulose degradation capacity microbes, decreased nutrient absorption efficiency and increased potential pathogenic bacteria. To reduce the risk of maladaptive factors due to captive conservation, the proportion of dietary fibers should be appropriately increased to help Père David's deer maintain a stable and health intestinal flora under artificial environment. Intestinal health is a critical factor regulating health, and the dynamic equilibrium between intestinal bacteria and the body affects digestion, metabolism, nutrient absorption, and immunity (Nicholson et al., 2012; Ottman et al., 2012; Zhang et al., 2023). To improve captive breeding strategies, more studies are still required to find ideal ways of dietary choices similar to wild, which will play an important role in the conservation of Père David's deer. Moreover, the original living environment, host evolutionary history, and dietary habits also affect the gut microbiota composition of deer (Wang et al., 2023). Nonetheless, further exploration is still necessary to ensure taxonomic accuracy due to the low resolution of amplicon sequencing.

The analysis of the neutral model provides new insights into the stochastic assembly process of gut microbiota communities. The neutral theory of biodiversity serves as a mechanistic model for predicting species coexistence and biodiversity patterns within ecological communities (O'Dwyer et al., 2015). Initially developed to predict communities of large organisms like animals and plants, it has recently been applied to the ecology of gut microbial communities (Heys et al., 2020; Li et al., 2022b; Zhu et al., 2022). According to the neutral theory, stochastic processes—independent of host traits—play significant roles in shaping the composition of microbial communities within an individual host. Previous studies have demonstrated that the gut microbiomes of Drosophila and Cotton Bollworm align well with the predictions of the neutral model (Adair et al., 2018; Li et al., 2022b). Similarly, neutral processes also dominated the gut microbial community assembly in Atlantic Salmon, *Salmo salar* (Heys et al., 2020). In the present study, six deer species exhibited a high relative contribution of neutral processes in the assembly of their gut microbial communities, with Père David's deer showing the highest relative involvement. This may be attributed to the fact that Père David's deer have been kept in captivity for shorter periods compared to other species. Studies have also shown that captivity reduces the stochastic processes involved in the assembly of gut microbiota communities in white-lipped deer (Li et al., 2022a). This could explain why restoring Père David's deer populations in the wild is challenging; their wild populations remain smaller than those of other deer species. This study highlights that neutral processes are predominant in the assembly of gut microbiota communities across different deer species, particularly in Père David's deer. Although the Neutral Community Model (NCM) is well applied, some deterministic factors on the composition of gut microbiota cannot be ignored, such as host genetics, dietary habits and original living environment. The host genes play a crucial role in shaping the composition and structure of the gut microbiome. The gut microbiome co-evolve with the host and can be stably transmitted to subsequent generations. Previous studies on inbred mice have shown that the gut microbiome is primarily transmitted through vertical transmission (Moeller et al., 2018). Furthermore, studies on hybrid offspring of sika deer (*Cervus nippon*) and elk (*Cervus elaphus*) have shown that the rumen microbiome differs from that of their parents, suggesting a significant effect of host genetics on the rumen microbiome may stem from vertical transmission (Li et al., 2016). These results demonstrated that host genetics play an important role in the formation of gut microbiome. Additionally, dietary differences in different living conditions, including seasonal changes, geographical locations, and captivity, can also cause changes in gut microbiota. Studies on white-lipped deer have shown that the diversity and richness of the gut microbiota in the grassy season was higher than those of the withering season (You et al., 2022). For the white-lipped deer population, sufficient and diverse fresh plant-derived food is available in the grassy season; however, food resources and choices are relatively limited in the withering season due to severe weather conditions. The results indicate that white-lipped deer had higher abundances of Firmicutes, Patescibacteria, and Bacteroidota in the grassy season, and higher abundances of Proteobacteria and Actinobacteria in the withering season. Furthermore, diet changes due to geographical locations can also affect the composition and structure of gut microbiota. The intestinal flora compositions of forest musk deer from Sichuan (subtropical monsoon climate) and Qinghai (highland continental climate, higher latitude, and lower temperature than Sichuan) differ significantly (Liu et al., 2019). Moreover, the Père David's deer populations living in Beijing (semihumid monsoon climate, higher latitude) and Shishou (subtropical monsoon climate, and higher temperature than Beijing) harbor very different gut microbiota, with the gut microbiota of deer in Beijing exhibiting a higher Firmicutes/Bacteroidota ratio than that of deer in Shishou (Zhang et al., 2018). Besides, comparative analysis of several studies on captive and wild deer revealed that the gut microbiota structure is very different. The relative abundance of Firmicutes is significantly higher in wild deer species than in captive ones, while the relative abundance of Bacteroidetes exhibits an opposite trend (Wang et al., 2023). The difference in gut microbiota between wild deer and captive deer populations may be due to the fact that wild deer species can obtain a diverse high fiber diet, while captive deer species mainly consume formula diets containing high starch, carbohydrate, and protein concentrations. Notably, the relative abundance of Proteobacteria in captive Sika deer and White-lipped deer is higher than that in wild Sika deer. This further indicates the impact of artificially formulated diets on the gut microbiota structure of captive deer species. Therefore, the results reveal that although stochastic processes were dominate in different deer species, deterministic processes such as host genetics, dietary habits and living environment were also found to play a crucial role in the assembly of gut microbiota.

In this study, we investigated the impact of gut microbes on the functional pathways of various deer species. Our comparative analysis of intestinal bacteria functions across different deer revealed significant distinctions in their functional composition, closely linked to the predominant microbial species in each deer. Using PICRUST2 analysis, we found marked differences in the intestinal flora's functional composition among the deer (adonis *P* < 0.05). The LEfSe differential pathway analysis and Sankey diagram demonstrated that Sika deer and Père David's deer had a higher number of EC-pathways compared to other deer species. Functional analysis based on the KEGG database indicated that intestinal microbiota could affect fatty acid and lipid biosynthesis, cofactor, carrier, and vitamin biosynthesis, amino acid biosynthesis, secondary metabolite biosynthesis, and other biosynthesis pathways, as well as superpathways of amino acid metabolism, further indicating that intestinal microbiota have a certain regulatory effect on the biosynthesis and substance metabolism. The most abundant branched-chain amino acids (BCAA), valine, isoleucine, and leucine are essential amino acids synthesized by plants, fungi, and bacteria, especially members of the gut microbiota. They play a crucial role in maintaining homeostasis in mammals by regulating protein synthesis, glucose and lipid metabolism, liver cell proliferation, and immunity (Tajiri and Shimizu, 2018). BCAA catabolism is crucial for controlling thermogenesis in brown adipose tissue. It occurs in mitochondria through the SLC25A44 transporters and helps improve metabolic status (Yoneshiro et al., 2019). In addition, supplementation of mice with a mixture of BCAAs promotes a healthy microbiota with an increase in Akkermansia and Bifidobacterium and a decrease in Enterobacteriaceae (Yang et al., 2016b). The gut microbiota is a modulator of BCAA levels, as it can both produce and use BCAAs. Prevotella, which has high relative abundance in RT, can degrade hemicellulose, pectin, and simpler carbohydrates such as those expected in fruits and low complexity fiber resources to produce BCAAs (Russell and Baldwin, 1979). Moreover, the genera found in our study, such as Clostridium, Ruminococcus, and Roseburia, can degrade fibers to produce SCFAs and organic acids (Koh et al., 2016). SCFA mainly includes propionate, butyrate, and acetate, which are the main anions in the intestine and can be rapidly absorbed by colonic epithelial cells. Propionic acid is mainly consumed by the liver for gluconeogenesis; Butyrate is the preferred energy source for colon cells; A large amount of acetate enters the systemic circulation for lipid generation (Pomare et al., 1985; Scott et al., 2008). Studies on the gut microbiota of wild and captive Alpine Musk Deer shown that more SCFAs can increase rate of nutrient uptake from the food in wild deer population, and SCFAs are thought to be responsible for ~50–70% of ruminant energy supply (Bergman, 1990; Sun et al., 2019b). Succinivibrio, a genus found in high abundance in Père David's deer, plays a role in various metabolic and biosynthetic pathways. This starch-degrading bacterium primarily produces acetate and succinate. Its high abundance might be associated with Père David's deer consuming diets rich in starch, such as grains and legumes. Similarly, a study by Tariq Shah et al. on yak fecal microbiota reported that the high-concentrate diet group showed a significantly higher abundance of Succinivibrio compared to the natural grazing group consuming diverse herbage (Shah et al., 2022). Additionally, Paenibacillus is another crucial player in the biosynthetic and metabolic pathways in Père David's deer. This genus produces valuable molecules, including exo-polysaccharides (EPS) and enzymes such as amylases, cellulases, hemicellulases, lipases, and pectinases, which can aid digestion and absorption (Grady et al., 2016). Our correlation heatmap analysis of the top 50 genera and pathways also identified Pseudomonas as a participant in these pathways in both PD and RT. *Pseudomonas aeruginosa*, a species within the Pseudomonas genus, is a Gram-negative bacterium known for its role as an opportunistic pathogen. As a common opportunistic pathogen, *P. aeruginosa* is highly prone to chronic infection and is nearly impossible to eradicate, particularly due to virulence factors and adaptive mutations (Jin et al., 2024). Based on our analysis and the observed abundance of Proteobacteria (Figure 2B), we speculate that Père David's deer may be susceptible to enteritis or diarrheal diseases. Consequently, we recommend increased focus on gastrointestinal health in captive animals, particularly endangered species like Père David's deer.

In conclusion, we conducted a thorough examination of the fecal microbiota, neutral model, and microbiota function across different groups of captive deer using amplicon sequencing and multi-statistical analysis. We found that the variations in microbial community composition, function, and community assembly are not only related to neutral processes, but also to the unique genetic background, dietary preferences, and living environment of each host species. However, there are several limitations to this study that should be addressed: (i) the sample size of captive deer should be increased; (ii) the sample size of wild deer should also be expanded; and (iii) advanced technologies such as metagenomics or metatranscriptomics should be utilized to delve deeper into microbial functional genes, especially those of pathogens. In summary, this study on fecal bacterial microbiota in various deer groups offers valuable insights into microbial diversity and provides theoretical knowledge beneficial to the conservation efforts of endangered species.

## Data Availability

The datasets presented in this study can be found in online repositories. The names of the repository/repositories and accession number(s) can be found at: https://www.ncbi.nlm.nih.gov/, accession number PRJNA1195701.

## References

[B1] Adair K. L. Wilson M. Bost A. Douglas A. E. (2018). Microbial community assembly in wild populations of the fruit fly *Drosophila melanogaster*. ISME J. 12, 959–972. 10.1038/s41396-017-0020-x 29358735 PMC5864213

[B2] Adak A. Khan M. R. (2019). An insight into gut microbiota and its functionalities. Cell. Mol. Life Sci. 76, 473–493. 10.1007/s00018-018-2943-4 30317530 PMC11105460

[B3] Andoh A. (2016). Physiological role of gut microbiota for maintaining human health. Digestion 93, 176–181. 10.1159/000444066 26859303

[B4] Bäckhed F. Ley R. E. Sonnenburg J. L. Peterson D. A. Gordon J. I. (2005). Host-bacterial mutualism in the human intestine. Science 307, 1915–1920. 10.1126/science.1104816 15790844

[B5] Bahrndorff S. Alemu T. Alemneh T. Lund Nielsen J. (2016). The microbiome of animals: implications for conservation biology. Int. J. Genom. 2016:5304028. 10.1155/2016/5304028 27195280 PMC4852354

[B6] Bergman E. N. (1990). Energy contributions of volatile fatty acids from the gastrointestinal tract in various species. Physiol. Rev. 70, 567–590. 10.1152/physrev.1990.70.2.567 2181501

[B7] Bolyen E. Rideout J. R. Dillon M. R. Bokulich N. A. Abnet C. C. Al-Ghalith G. A.. (2019). Reproducible, interactive, scalable and extensible microbiome data science using QIIME 2. Nat. Biotechnol. 37, 852–857. 10.1038/s41587-019-0209-9 31341288 PMC7015180

[B8] Burns A. R. Stephens W. Z. Stagaman K. Wong S. Rawls J. F. Guillemin K.. (2016). Contribution of neutral processes to the assembly of gut microbial communities in the zebrafish over host development. ISME J. 10, 655–664. 10.1038/ismej.2015.142 26296066 PMC4817674

[B9] Callahan B. J. McMurdie P. J. Rosen M. J. Han A. W. Johnson A. J. Holmes S. P. (2016). DADA2: high-resolution sample inference from Illumina amplicon data. Nat. Methods 13, 581–583. 10.1038/nmeth.3869 27214047 PMC4927377

[B10] Douglas G. M. Maffei V. J. Zaneveld J. R. Yurgel S. N. Brown J. R. Taylor C. M.. (2020). PICRUSt2 for prediction of metagenome functions. Nat. Biotechnol. 38, 685–688. 10.1038/s41587-020-0548-6 32483366 PMC7365738

[B11] Ezenwa V. O. Gerardo N. M. Inouye D. W. Medina M. Xavier J. B. (2012). Microbiology. Animal behavior and the microbiome. Science 338, 198–199. 10.1126/science.1227412 23066064

[B12] Fitzgerald S. F. Mitchell M. C. Holmes A. Allison L. Chase-Topping M. Lupolova N.. (2023). Prevalence of Shiga toxin-producing *Escherichia coli* O157 in Wild Scottish deer with high human pathogenic potential. Animals 13:2795. 10.3390/ani13172795 37685059 PMC10486872

[B13] Flint H. J. Bayer E. A. Rincon M. T. Lamed R. White B. A. (2008). Polysaccharide utilization by gut bacteria: potential for new insights from genomic analysis. Nat. Rev. Microbiol. 6, 121–131. 10.1038/nrmicro1817 18180751

[B14] Grady E. N. MacDonald J. Liu L. Richman A. Yuan Z. C. (2016). Current knowledge and perspectives of *Paenibacillus*: a review. Microb. Cell Fact. 15:203. 10.1186/s12934-016-0603-7 27905924 PMC5134293

[B15] Heys C. Cheaib B. Busetti A. Kazlauskaite R. Maier L. Sloan W. T.. (2020). Neutral processes dominate microbial community assembly in Atlantic Salmon, Salmo salar. Appl. Environ. Microbiol. 86:e02283-19. 10.1128/AEM.02283-19 32033945 PMC7117918

[B16] Holmes E. Li J. V. Marchesi J. R. Nicholson J. K. (2012). Gut microbiota composition and activity in relation to host metabolic phenotype and disease risk. Cell Metab. 16, 559–564. 10.1016/j.cmet.2012.10.007 23140640

[B17] Honore-Bouakline S. Vincensini J. P. Giacuzzo V. Lagrange P. H. Herrmann J. L. (2003). Rapid diagnosis of extrapulmonary tuberculosis by PCR: impact of sample preparation and DNA extraction. J. Clin. Microbiol. 41, 2323–2329. 10.1128/JCM.41.6.2323-2329.2003 12791844 PMC156509

[B18] Hooper L. V. (2004). Bacterial contributions to mammalian gut development. Trends Microbiol. 12, 129–134. 10.1016/j.tim.2004.01.001 15001189

[B19] Hubbell S. P. (2001). The Unified Neutral Theory of Biodiversity and Biogeography, 32. Princeton, NJ: Princeton University Press.

[B20] Jandhyala S. M. Talukdar R. Subramanyam C. Vuyyuru H. Sasikala M. Nageshwar Reddy D. (2015). Role of the normal gut microbiota. World J. Gastroenterol. 21, 8787–8803. 10.3748/wjg.v21.i29.8787 26269668 PMC4528021

[B21] Jeong M. Y. Jang H. M. Kim D. H. (2019). High-fat diet causes psychiatric disorders in mice by increasing Proteobacteria population. Neurosci. Lett. 698, 51–57. 10.1016/j.neulet.2019.01.006 30615977

[B22] Jiang Z. Harris R. B. (2016). Elaphurus davidianus. The IUCN Red List of Threatened Species 2016:e.T7121A22159785. 10.2305/IUCN.UK.2016-2.RLTS.T7121A22159785.en32055894

[B23] Jiang Z. Yu C. Feng Z. (2000). Reintroduction and recovery of Pere David's deer in China. Wildlife Soc. Bull. 28, 681–687. 10.2307/3783620

[B24] Jin X. Zhang C. Lin S. Gao T. Qian H. Qu L.. (2024). Pec 1 of *Pseudomonas aeruginosa* inhibits bacterial clearance of host by blocking autophagy in macrophages. ACS Infect. Dis. 10, 2741–2754. 10.1021/acsinfecdis.4c00096 39047963

[B25] Keqing C. (1985). On the reasons of extinction of the wild mi-deer in china. Zool. Res. 6, 111–115.

[B26] Kers J. G. Velkers F. C. Fischer E. A. J. Hermes G. D. A. Stegeman J. A. Smidt H. (2018). Host and environmental factors affecting the intestinal microbiota in chickens. Front. Microbiol. 9:235. 10.3389/fmicb.2018.00235 29503637 PMC5820305

[B27] Koh A. De Vadder F. Kovatcheva-Datchary P. Bäckhed F. (2016). From dietary fiber to host physiology: short-chain fatty acids as key bacterial metabolites. Cell 165, 1332–1345. 10.1016/j.cell.2016.05.041 27259147

[B28] Kotloff K. L. Nataro J. P. Blackwelder W. C. Nasrin D. Farag T. H. Panchalingam S.. (2013). Burden and aetiology of diarrhoeal disease in infants and young children in developing countries (the Global Enteric Multicenter Study, GEMS): a prospective, case-control study. Lancet 382, 209–222. 10.1016/S0140-6736(13)60844-2 23680352

[B29] Li B. Gao H. Song P. Liang C. Jiang F. Xu B.. (2022a). Captivity shifts gut microbiota communities in white-lipped deer (*Cervus albirostris*). *Animals* 12:431. 10.3390/ani12040431 35203139 PMC8868073

[B30] Li S. Tang R. Yi H. Cao Z. Sun S. Liu T. X.. (2022b). Neutral processes provide an insight into the structure and function of gut microbiota in the cotton bollworm. Front. Microbiol. 13:849637. 10.3389/fmicb.2022.849637 35591990 PMC9113526

[B31] Li Z. Wright A. G. Si H. Wang X. Qian W. Zhang Z.. (2016). Changes in the rumen microbiome and metabolites reveal the effect of host genetics on hybrid crosses. Environ. Microbiol. Rep. 8, 1016–1023. 10.1111/1758-2229.12482 27717170

[B32] Liu X. Li D. Q. Zhao W. Yu D. Cheng J. G. Luo Y.. (2019). “Sequencing and analysis of gut microbiota in forest musk deer from Qinghai and Sichuan,” in BIBE The Third International Conference on Biological Information and Biomedical Engineering, VDE, Hangzhou, China, 1–7.

[B33] Malmuthuge N. Guan L. L. (2017). Understanding host-microbial interactions in rumen: searching the best opportunity for microbiota manipulation. J. Anim. Sci. Biotechnol. 8:8. 10.1186/s40104-016-0135-3 28116074 PMC5244612

[B34] Méndez-Salazar E. O. Ortiz-López M. G. Granados-Silvestre M. Palacios-González B. Menjivar M. (2018). Corrigendum: Altered gut microbiota and compositional changes in firmicutes and proteobacteria in Mexican undernourished and obese children. Front. Microbiol. 9:2693. 10.3389/fmicb.2018.02693 30386323 PMC6198253

[B35] Moeller A. H. Peeters M. Ndjango J. B. Li Y. Hahn B. H. Ochman H. (2013). Sympatric chimpanzees and gorillas harbor convergent gut microbial communities. Genome Res. 23, 1715–1720. 10.1101/gr.154773.113 23804402 PMC3787267

[B36] Moeller A. H. Suzuki T. A. Phifer-Rixey M. Nachman M. W. (2018). Transmission modes of the mammalian gut microbiota. Science 362, 453–457. 10.1126/science.aat7164 30361372

[B37] Muegge B. D. Kuczynski J. Knights D. Clemente J. C. González A. Fontana L.. (2011). Diet drives convergence in gut microbiome functions across mammalian phylogeny and within humans. Science 332, 970–974. 10.1126/science.1198719 21596990 PMC3303602

[B38] Ndeh D. Gilbert H. J. (2018). Biochemistry of complex glycan depolymerisation by the human gut microbiota. FEMS Microbiol. Rev. 42, 146–164. 10.1093/femsre/fuy002 29325042

[B39] Nicholson J. K. Holmes E. Kinross J. Burcelin R. Gibson G. Jia W.. (2012). Host-gut microbiota metabolic interactions. Science 336, 1262–1267. 10.1126/science.1223813 22674330

[B40] Nyaoke A. C. Navarro M. A. Fresneda K. Diab S. S. Moore J. Lyras D.. (2020). *Paeniclostridium (Clostridium) sordellii*-associated enterocolitis in 7 horses. J. Vet. Diagn. Invest. 32, 239–245. 10.1177/1040638720903738 32052697 PMC7081492

[B41] O'Dwyer J. P. Kembel S. W. Sharpton T. J. (2015). Backbones of evolutionary history test biodiversity theory for microbes. Proc. Natl. Acad. Sci. U.S.A. 112, 8356–8361. 10.1073/pnas.1419341112 26106159 PMC4500224

[B42] Oksanen J. Kindt R. Legendre P. O'Hara B. Stevens M. H. H. Oksanen M. J.. (2007). The vegan package. Commun. Ecol. Package 10:719.

[B43] Ottman N. Smidt H. de Vos W. M. Belzer C. (2012). The function of our microbiota: who is out there and what do they do? Front. Cell Infect. Microbiol. 2:104. 10.3389/fcimb.2012.00104 22919693 PMC3417542

[B44] Pomare E. W. Branch W. J. Cummings J. H. (1985). Carbohydrate fermentation in the human colon and its relation to acetate concentrations in venous blood. J. Clin. Invest. 75, 1448–1454. 10.1172/JCI111847 3998144 PMC425482

[B45] Prentice M. B. Gilbertson M. L. J. Storm D. J. Turner W. C. Walsh D. P. Pinkerton M. E.. (2024). Metagenomic sequencing sheds light on microbes putatively associated with pneumonia-related fatalities of white-tailed deer (*Odocoileus virginianus*). *Microb. Genom*. 10:001214. 10.1099/mgen.0.001214 38536208 PMC10995629

[B46] Qasim M. Wrage M. Nüse B. Mattner J. (2022). Shigella outer membrane vesicles as promising targets for vaccination. Int. J. Mol. Sci. 23:994. 10.3390/ijms23020994 35055181 PMC8781765

[B47] Qiu H. Chen F. Leng X. Fei R. Wang L. (2014). Toxinotyping of *Clostridium perfringens* fecal isolates of reintroduced Père David's deer (*Elaphurus davidianus*) in China. J. Wildl. Dis. 50, 942–945. 10.7589/2013-05-125 25050802

[B48] Quast C. Pruesse E. Yilmaz P. Gerken J. Schweer T. Yarza P.. (2013). The SILVA ribosomal RNA gene database project: improved data processing and web-based tools. Nucleic Acids Res. 41, D590–596. 10.1093/nar/gks1219 23193283 PMC3531112

[B49] Russell J. B. Baldwin R. L. (1979). Comparison of maintenance energy expenditures and growth yields among several rumen bacteria grown on continuous culture. Appl. Environ. Microbiol. 37, 537–543. 10.1128/aem.37.3.537-543.1979 16345359 PMC243251

[B50] Scott K. P. Martin J. C. Mrazek J. Flint H. J. (2008). Transfer of conjugative elements from rumen and human Firmicutes bacteria to *Roseburia inulinivorans*. Appl. Environ. Microbiol. 74, 3915–3917. 10.1128/AEM.02807-07 18456856 PMC2446557

[B51] Segata N. Izard J. Waldron L. Gevers D. Miropolsky L. Garrett W. S.. (2011). Metagenomic biomarker discovery and explanation. Genome Biol. 12:R60. 10.1186/gb-2011-12-6-r60 21702898 PMC3218848

[B52] Shah T. Ding L. Ud Din A. Hassan F. U. Ahmad A. A. Wei H.. (2022). Differential effects of natural grazing and feedlot feeding on yak fecal microbiota. Front. Vet. Sci. 9:791245. 10.3389/fvets.2022.791245 35529830 PMC9074760

[B53] Sloan W. T. Lunn M. Woodcock S. Head I. M. Nee S. Curtis T. P. (2006). Quantifying the roles of immigration and chance in shaping prokaryote community structure. Environ. Microbiol. 8, 732–740. 10.1111/j.1462-2920.2005.00956.x 16584484

[B54] Sun C. H. Liu H. Y. Liu B. Yuan B. D. Lu C. H. (2019a). Analysis of the gut microbiome of wild and captive Père David's deer. Front. Microbiol. 10:2331. 10.3389/fmicb.2019.02331 31636626 PMC6787558

[B55] Sun Y. Sun Y. Shi Z. Liu Z. Zhao C. Lu T.. (2019b). Gut microbiota of wild and captive Alpine Musk deer (*Moschus chrysogaster*). *Front. Microbiol*. 10:3156. 10.3389/fmicb.2019.03156 32038587 PMC6985557

[B56] Sun Y. Zhang S. Nie Q. He H. Tan H. Geng F.. (2023). Gut firmicutes: relationship with dietary fiber and role in host homeostasis. Crit. Rev. Food Sci. Nutr. 63, 12073–12088. 10.1080/10408398.2022.2098249 35822206

[B57] Tajiri K. Shimizu Y. (2018). Branched-chain amino acids in liver diseases. Transl. Gastroenterol. Hepatol. 3:47. 10.21037/tgh.2018.07.06 30148232 PMC6088198

[B58] Tap J. Mondot S. Levenez F. Pelletier E. Caron C. Furet J. P.. (2009). Towards the human intestinal microbiota phylogenetic core. Environ. Microbiol. 11, 2574–2584. 10.1111/j.1462-2920.2009.01982.x 19601958

[B59] Waite D. W. Eason D. K. Taylor M. W. (2014). Influence of hand rearing and bird age on the fecal microbiota of the critically endangered kakapo. Appl. Environ. Microbiol. 80, 4650–4658. 10.1128/AEM.00975-14 24837385 PMC4148800

[B60] Wang L. Ding J. Yang Z. Chen H. Yao R. Dai Q.. (2019). Père David's deer gut microbiome changes across captive and translocated populations: Implications for conservation. Evol. Appl. 12, 622–635. 10.1111/eva.12743 30828378 PMC6383733

[B61] Wang Y. Xu B. Chen H. Yang F. Huang J. Jiao X.. (2023). Environmental factors and gut microbiota: toward better conservation of deer species. Front. Microbiol. 14:1136413. 10.3389/fmicb.2023.1136413 36960286 PMC10027939

[B62] Yan L. Lv Z. Z. An S. Xing K. Wang Z. G. Lv M. B.. (2021). Effects of rearing system and narasin on growth performance, gastrointestinal development, and gut microbiota of broilers. Poult. Sci. 100:100840. 10.1016/j.psj.2020.10.073 33531152 PMC7936129

[B63] Yang D. Song Y. Ma J. Li P. Zhang H. Price M. R.. (2016a). Stepping-stones and dispersal flow: establishment of a meta-population of Milu (*Elaphurus davidianus*) through natural re-wilding. Sci. Rep. 6:27297. 10.1038/srep27297 27272326 PMC4895148

[B64] Yang Z. Huang S. Zou D. Dong D. He X. Liu N.. (2016b). Metabolic shifts and structural changes in the gut microbiota upon branched-chain amino acid supplementation in middle-aged mice. Amino Acids 48, 2731–2745. 10.1007/s00726-016-2308-y 27539648

[B65] Yapin M. (1986). Report on diagnosing and controlling braxy of Sika deer. J. Jilin Agric. Univ.

[B66] Yeoman C. J. White B. A. (2014). Gastrointestinal tract microbiota and probiotics in production animals. Annu. Rev. Anim. Biosci. 2, 469–486. 10.1146/annurev-animal-022513-114149 25384152

[B67] Yoneshiro T. Wang Q. Tajima K. Matsushita M. Maki H. Igarashi K.. (2019). BCAA catabolism in brown fat controls energy homeostasis through SLC25A44. Nature 572, 614–619. 10.1038/s41586-019-1503-x 31435015 PMC6715529

[B68] You Z. Deng J. Liu J. Fu J. Xiong H. Luo W.. (2022). Seasonal variations in the composition and diversity of gut microbiota in white-lipped deer (*Cervus albirostris*). *PeerJ* 10:e13753. 10.7717/peerj.13753 35873913 PMC9302429

[B69] Zhang B. Shi M. Xu S. Zhang H. Li Y. Hu D. (2023). Analysis on changes and influencing factors of the intestinal microbiota of Alpine Musk deer between the place of origin and migration. Animals 13:3791. 10.3390/ani13243791 38136828 PMC10740494

[B70] Zhang M. Shi M. Fan M. Xu S. Li Y. Zhang T.. (2018). Comparative analysis of gut microbiota changes in Père David's deer populations in Beijing Milu Park and Shishou, Hubei Province in China. Front. Microbiol. 9:1258. 10.3389/fmicb.2018.01258 29946310 PMC6005820

[B71] Zhao C. Liu L. Gao L. Bai L. (2022). A comprehensive comparison of fecal microbiota in three ecological bird groups of raptors, waders, and waterfowl. Front. Microbiol. 13:919111. 10.3389/fmicb.2022.919111 36003944 PMC9393522

[B72] Zhou L. Huo X. Liu B. Wu H. Feng J. (2020). Comparative analysis of the gut microbial communities of the Eurasian Kestrel (*Falco tinnunculus*) at different developmental stages. Front. Microbiol. 11:592539. 10.3389/fmicb.2020.592539 33391209 PMC7775371

[B73] Zhu J. Li H. Jing Z. Z. Zheng W. Luo Y. R. Chen S. X.. (2022). Robust host source tracking building on the divergent and non-stochastic assembly of gut microbiomes in wild and farmed large yellow croaker. Microbiome 10:18. 10.1186/s40168-021-01214-7 35081990 PMC8790850

[B74] Zhu Y. Wang C. Li F. (2015). Impact of dietary fiber/starch ratio in shaping caecal microbiota in rabbits. Can. J. Microbiol. 61, 771–784. 10.1139/cjm-2015-0201 26361938

