# Supplementary material for: Comparative analysis of the fecal microbiota in Père David's deer and five other captive deer species
Source: Front Microbiol. 2025 Mar 26;16:1547348. doi: 10.3389/fmicb.2025.1547348 (PMC11979286; doi:10.3389/fmicb.2025.1547348)
Supplement: Supplementary file 1 [file Data_Sheet_1.docx]

Supplementary Material


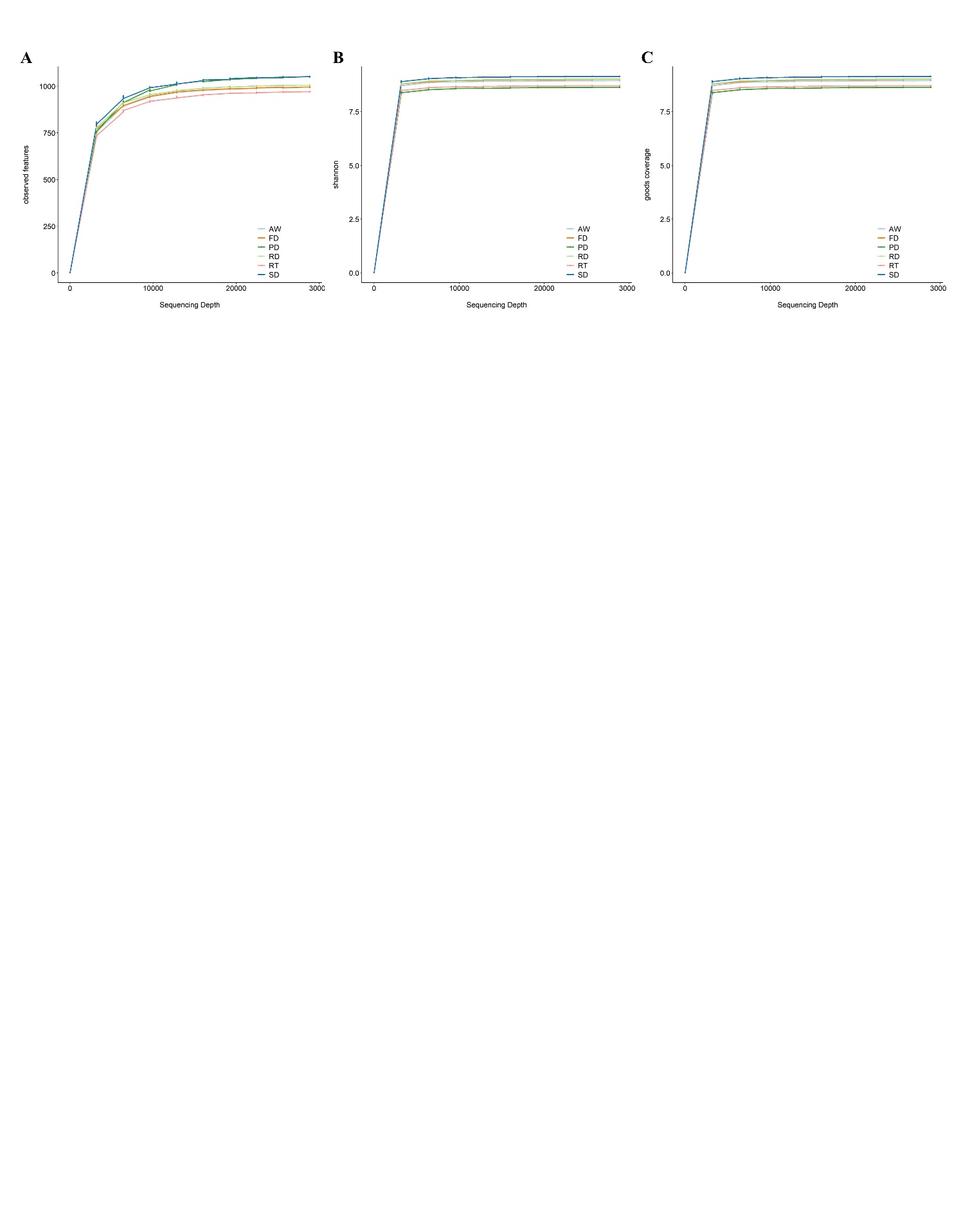
Supplementary Figure 1. The rarefaction (A), Shannon-Wiener curves (B) and goods coverage for the groups of six deer species.


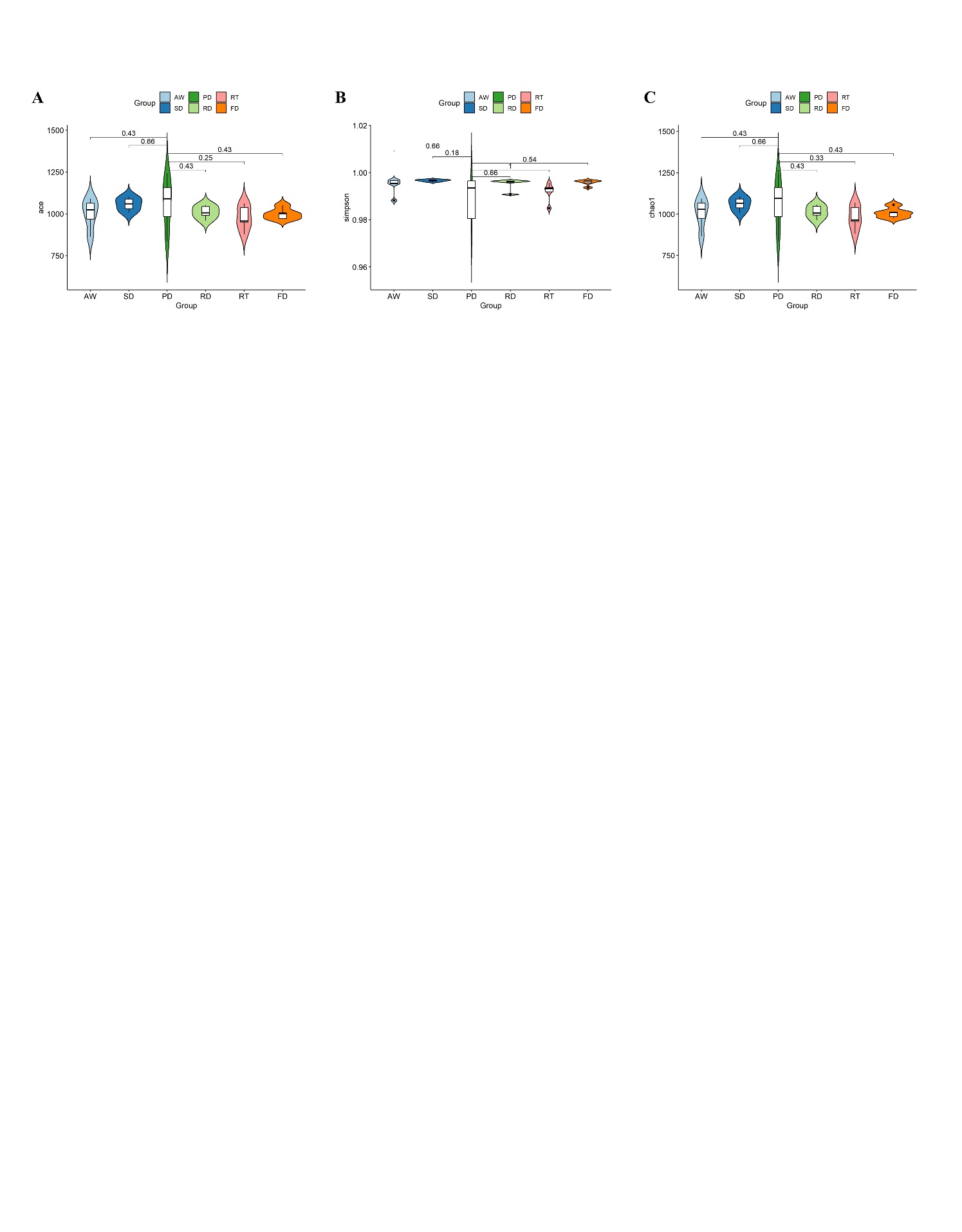


Supplementary Figure 2. Violin plots depicting differences in ASV richness among difference deer species using ace index (A), simpson index (B) and chao1 index (C).


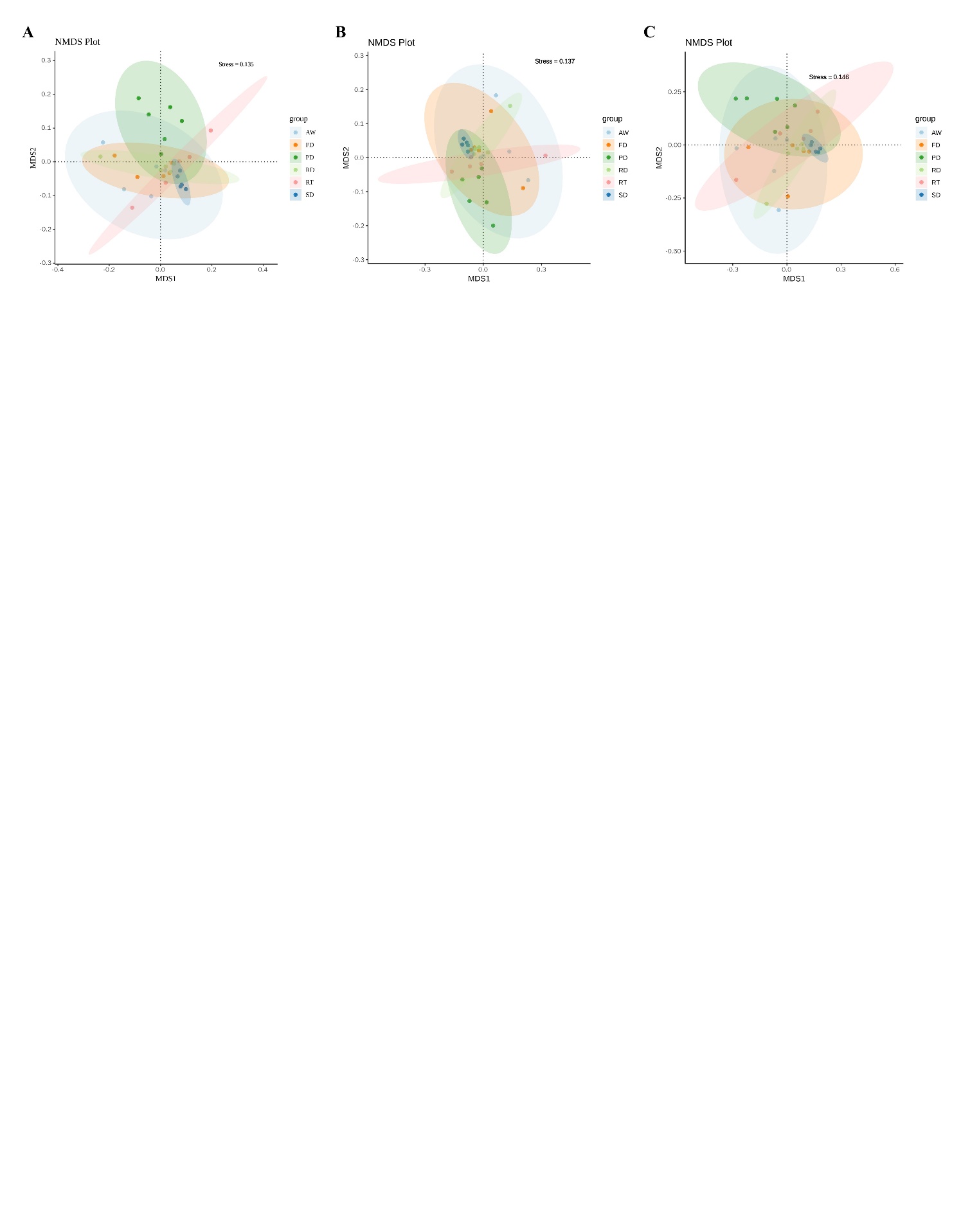


Supplementary Figure 3. Nonmetric multidimensional scaling (NMDS) ordinations based on Bray–Curtis distances and Bonferroni based on ANOSIM in fecal microbiota of six deer species. COG (adonis: *p*-value=0.022) (A), EC (adonis: *p*-value=0.012) (B), KO (adonis: *p*-value=0.016) (C).
